# Correlation of serum ferritin and D-Dimer levels with clinical outcomes among hospitalized Indian patients

**DOI:** 10.6026/973206300221643

**Published:** 2026-03-31

**Authors:** Pankaj Kumar Jain, Mahavir Raghunath Mundra, Lalan Pratap Singh

**Affiliations:** 1 Department of General Medicine, Nandkumar Singh Chouhan Government Medical College, Khandwa, Madhya Pradesh, India; 2 Department of Biochemistry, N.K.P. Salve Institute of Medical Sciences & Research Centre and Lata Mangeshkar hospital, Nagpur, Maharashtra, India; 3 Department of General Medicine, Government Medical College, Satna, Madhya Pradesh, India

**Keywords:** Serum ferritin, D-dimer, risk stratification, intensive care, mortality prediction, biomarkers

## Abstract

Serum ferritin and D-dimer are established biomarkers reflecting inflammatory activity and coagulation disturbances, both of which are linked to adverse outcomes in hospitalized individuals. Therefore, it is of interest to determine the relationship between admission ferritin and D-dimer levels and unfavorable clinical outcomes in hospitalized adults. In this cross-sectional observational analysis of 200 patients, biomarker levels obtained within 24 hours of admission were evaluated in relation to intensive care requirement and in hospital mortality using correlation analysis, multivariable logistic regression and receiver operating characteristic curve analysis. Thus, serum ferritin and D-dimer levels, when assessed together at admission, provide valuable predictive insight into adverse clinical outcomes, offering potential for early prognostic evaluation and improved risk stratification in hospitalized patients.

## Background:

Systemic inflammation and activation of coagulation pathways are key contributors to poor outcomes in patients admitted with severe medical illnesses. Serum ferritin, an acute phase reactant, serves as a marker of inflammatory burden and has been extensively investigated as a prognostic indicator during the COVID-19 era and other infectious conditions [1, 2]. Elevated ferritin levels have been linked with increased disease severity, cytokine-mediated injury, organ dysfunction and mortality in multiple clinical studies [3, 4]. D-dimer, a degradation product of cross-linked fibrin, reflects enhanced coagulation and fibrinolytic activity. Evidence from studies published since 2020 indicates that elevated D-dimer levels at the time of hospital admission are associated with thrombotic complications, intensive care requirement and increased mortality risk [5, 6]. The presence of hypercoagulability and microvascular thrombosis further underscores the prognostic relevance of D-dimer in critically ill populations [7]. Emerging literature suggests that combined evaluation of inflammatory and coagulation markers may provide superior prognostic information compared to single biomarkers alone [8]. Nevertheless, data examining the simultaneous relationships of ferritin and D-dimer with outcomes across heterogeneous hospitalized populations remain limited [9]. Therefore, it is of interest to describe the combined prognostic value of serum ferritin and D-dimer levels at admission, as their assessment may enhance early risk stratification and improve clinical decision-making in hospitalized patients.

## Materials and Methods:

This cross-sectional observational study included 200 consecutively admitted adult patients at a tertiary care hospital during a defined study period. Serum ferritin and D-dimer concentrations were measured within 24 hours of admission using standardized laboratory techniques. Patients were monitored throughout hospitalization until discharge or death. Adverse clinical outcome was defined as ICU admission and/or in-hospital mortality. The study protocol received approval from the Institutional Ethics Committee and informed consent was obtained in accordance with ethical standards.

## Inclusion criteria:

[1] Age ≥18 years

[2] Hospitalization for acute medical illness

[3] Ferritin and D-dimer measured within 24 hours of admission

[4] Availability of complete laboratory and clinical data

## Exclusion criteria:

[1] Known hematologic malignancy or chronic iron overload disorder

[2] Recent major trauma or surgery within 7 days

[3] Pre-existing long-term anticoagulation therapy

[4] Incomplete data records

## Statistical analysis:

Continuous variables were presented as mean±standard deviation or median (interquartile range) depending on distribution. Group comparisons were performed using Student's t-test or Mann-Whitney U test, while categorical variables were evaluated using chi-square analysis. Associations between biomarkers and clinical outcomes were assessed using Spearman correlation. Multivariable logistic regression analysis was conducted to identify independent predictors of adverse outcomes. Predictive performance was evaluated using ROC curve analysis and statistical significance was set at p < 0.05.

## Results:

A total of 200 hospitalized patients were included in the study, of whom 60 (30%) experienced adverse clinical outcomes, defined as ICU admission and/or in-hospital mortality. Patients with adverse outcomes were significantly older than those without adverse outcomes (61.8±13.6 vs 52.3±14.1 years, p < 0.001), were more often male (70.0% vs 55.7%, p = 0.048), and had a greater comorbidity burden (1.9±1.1 vs 1.1±0.9, p < 0.001) (Table 1 - see PDF). With respect to laboratory findings, both serum ferritin and D-dimer levels were markedly elevated in patients with adverse outcomes. Median ferritin levels were 1180 ng/mL (IQR: 820-1650) in the adverse outcome group compared with 410 ng/mL (IQR: 250-580) in those without adverse outcomes (p < 0.001). Similarly, median D-dimer levels were substantially higher in patients with adverse outcomes [2950 ng/mL (IQR: 2100-4100) vs 820 ng/mL (IQR: 500-1150), p < 0.001] (Table 2 - see PDF). Spearman correlation analysis demonstrated significant positive associations between ferritin and adverse outcome (r = 0.52, p < 0.001), D-dimer and adverse outcome (r = 0.49, p < 0.001), and ferritin and D-dimer (r = 0.44, p < 0.001), indicating moderate correlations among these variables (Table 3 - see PDF). In multivariable logistic regression analysis, both log-transformed ferritin and log-transformed D-dimer remained independent predictors of adverse outcome after adjustment for age and comorbidity count. Log ferritin was associated with more than a twofold increase in odds of adverse outcome (adjusted OR 2.18, 95% CI 1.45-3.27, p < 0.001), while log D-dimer was associated with a 1.94-fold increase (95% CI 1.28-2.95, p = 0.002). Age (adjusted OR 1.04, 95% CI 1.01-1.07, p = 0.006) and comorbidity count (adjusted OR 1.36, 95% CI 1.10-1.69, p = 0.004) were also significant independent predictors. The final model showed acceptable discriminative performance, with an AUC of 0.78 (Table 4 - see PDF). As shown in Figure 1 (see PDF), median serum ferritin levels were significantly higher in patients with adverse clinical outcomes compared to those without adverse outcomes (p < 0.001). Figure 2 (see PDF) illustrates that median D-dimer levels were significantly elevated in patients with adverse outcomes, further supporting the association between elevated biomarkers and poor clinical outcomes.

## Discussion:

In this study, elevated serum ferritin levels were associated with adverse clinical outcomes, consistent with prior research linking hyperferritinemia to inflammatory dysregulation and organ injury [10, 11]. Excessive inflammatory activation and cytokine-mediated damage may explain the observed relationship between ferritin and poor prognosis [12]. Similarly, increased D-dimer concentrations were independently associated with unfavorable outcomes, supporting previous evidence that activation of coagulation pathways contributes to mortality risk in hospitalized patients [13, 14]. Persistent coagulation abnormalities have been correlated with prolonged hospital stay and higher complication rates [15]. The combined evaluation of ferritin and D-dimer improved prognostic discrimination compared to single-marker assessment, aligning with recent studies advocating multimarker risk stratification strategies [16, 17]. These findings reinforce the importance of integrating inflammatory and coagulation parameters in early clinical assessment. However, limitations including single-center design and the observational nature of the study must be acknowledged. The cross-sectional framework precludes definitive conclusions regarding causality between biomarker elevation and adverse outcomes. Larger prospective studies are required to establish standardized cut off values and confirm clinical applicability.

## Conclusion:

Admission serum ferritin and D-dimer levels are significantly correlated with adverse clinical outcomes in hospitalized patients. Both biomarkers independently predict ICU admission and in-hospital mortality and their combined assessment enhances prognostic accuracy. Routine evaluation of these accessible laboratory parameters may facilitate early risk stratification and support clinical decision- making.
